# Síndrome de Heyde: Estratégias Terapêuticas e Seguimento de Longo Prazo

**DOI:** 10.36660/abc.20200371

**Published:** 2021-06-25

**Authors:** Vitor Emer Egypto Rosa, Henrique Barbosa Ribeiro, João Ricardo Cordeiro Fernandes, Antonio de Santis, Guilherme Sobreira Spina, Milena Ribeiro Paixão, Lucas José Tachotti Pires, Marcelo Bettega, Tarso Augusto Duenhas Accorsi, Roney Orismar Sampaio, Flávio Tarasoutchi

**Affiliations:** 1 Instituto do Coração do Hospital das Clinicas Faculdade de Medicina Universidade de São Paulo São Paulo SP Brasil Instituto do Coração do Hospital das Clinicas da Faculdade de Medicina da Universidade de São Paulo, São Paulo, SP - Brasil

**Keywords:** Estenose Aórtica, Angiodisplasia, Hemorragia, Mortalidade, Cirurgia Torácica, Ecocardiografia/métodos

## Abstract

**Fundamentos:**

A síndrome de Heyde é a associação de estenose aórtica importante com episódio de sangramento gastrointestinal por lesões angiodisplásicas. Pouco é conhecido sobre os fatores associados a novos sangramentos e desfechos em longo prazo. Além disso, a maioria dos dados é restrita a relatos de casos e pequenas séries.

**Objetivo:**

Avaliar o perfil clínico, laboratorial e ecocardiográfico de pacientes com síndrome de Heyde submetidos a intervenção valvar ou tratamento medicamentoso.

**Métodos:**

Coorte prospectiva de 24 pacientes consecutivos entre 2005 e 2018. Foram avaliados dados clínicos, laboratoriais, ecocardiográficos e relacionados à intervenção valvar e a desfechos após o diagnóstico. Valor de p<0,05 foi considerado estatisticamente significante.

**Resultados:**

Metade dos 24 pacientes apresentou sangramento com necessidade de transfusão sanguínea na admissão. Angiodisplasias foram encontradas mais frequentemente no cólon ascendente (62%). Intervenção valvar (cirúrgica ou transcateter) foi realizada em 70,8% dos pacientes, e 29,2% foram mantidos em tratamento clínico. Novos episódios de sangramento ocorreram em 25% dos casos, e não houve diferença entre os grupos clínico e intervenção (28,6 vs. 23,5%, p=1,00; respectivamente). A mortalidade no seguimento de 2 e 5 anos foi de 16% e 25%, sem diferença entre os grupos (log-rank p = 0,185 e 0,737, respectivamente).

**Conclusões:**

Pacientes com síndrome de Heyde tiveram alta taxa de sangramento com necessidade de transfusão sanguínea na admissão, sugerindo ser uma doença grave e com risco elevado de mortalidade. Não encontramos diferenças entre os grupos submetidos ao tratamento clínico e à intervenção valvar em relação a taxas de ressangramento e mortalidade tardia.

## Introdução

A associação entre sangramento gastrointestinal por lesões angiodisplásicas e estenose aórtica importante é denominada síndrome de Heyde e foi descrita por Edward Heyde em 1958.^1^ Desde então, ainda há controvérsias sobre a patogênese de tal síndrome, e o único fator que comprovadamente predispõe a sangramentos nessa população é a presença da deficiência adquirida do fator de von Willebrand ( *von Willebrand factor* , vWF) decorrente do estresse hemodinâmico no orifício valvar aórtico estenótico.^2 - 6^

A síndrome de Heyde é descrita em 1,7% dos pacientes com estenose aórtica importante e, apesar de sua prevalência, pouco é conhecido sobre seus aspectos epidemiológicos, e a maioria das publicações relacionadas a essa entidade são relatos de casos e pequenas séries.^5^ A indicação de intervenção valvar nesses pacientes segue as diretrizes atuais e, apesar de não existirem recomendações específicas, alguns centros advogam intervenção para redução de sangramento independente da presença de sintomas ou complicadores.^7 - 9^ Assim, o objetivo deste estudo foi avaliar os aspectos clínicos, laboratoriais e ecocardiográficos, além da mortalidade e de complicações após intervenção valvar ou tratamento clínico, em uma série de casos de pacientes com síndrome de Heyde.

## Métodos

Trata-se de uma coorte prospectiva que avaliou pacientes com estenose aórtica importante em um centro terciário entre 2005 e 2018. A estenose aórtica importante foi definida como a presença de área valvar aórtica ≤ 1,0 cm^2^, gradiente transaórtico médio > 40 mmHg ou velocidade de pico > 4,0 m/s pelo ecocardiograma transtorácico.^9^ Pacientes com história de sangramento gastrointestinal e angiodisplasia documentada por colonoscopia e/ou endoscopia digestiva alta foram selecionados. Foram avaliados dados clínicos, laboratoriais, ecocardiográficos e relacionados ao tratamento indicado. Os critérios de exclusão foram: presença de outras condições que pudessem justificar o sangramento (como úlcera gástrica ou neoplasias), insuficiência aórtica importante ou valvopatia mitral primária anatomicamente importante. A indicação da intervenção valvar foi realizada de acordo com a escolha do médico assistente e seguindo as diretrizes vigentes.^9 , 10^ Para fins comparativos, dividimos os pacientes em dois grupos, de acordo com o tratamento escolhido (intervenção valvar vs. tratamento clínico medicamentoso). Avaliamos mortalidade e complicações perioperatórias em 30 dias e, após, através de contato telefônico. Este estudo foi aprovado pelo comitê de ética e pesquisa institucional, tendo sido solicitada a dispensa do termo de consentimento.

## Análise estatística

As variáveis contínuas e categóricas foram apresentadas como mediana (intervalo interquartil) e frequências ou porcentagens, respectivamente. A normalidade das variáveis foi avaliada com o teste de Kolmogorov-Smirnov. Variáveis contínuas foram analisadas através do teste de Mann-Whitney e variáveis categóricas através teste chi-quadrado ou teste exato de Fisher, conforme apropriado. A mortalidade em 5 anos foi avaliada utilizando teste Log-rank. Todos os testes foram bicaudais, e um valor de p < 0,05 foi utilizado para indicar significância estatística. O software SPSS versão 20 (IBM, Armonk, New York, USA) foi utilizado para analisar os dados.

## Resultados

Um total de 24 pacientes apresentou perfil para inclusão no estudo. As características basais da população estão descritas na Tabela 1 . A mediana etária foi de 77 [70-82] anos, 50% dos pacientes eram do sexo feminino, e encontramos uma alta prevalência de comorbidades como hipertensão arterial sistêmica (79,2%), diabetes (41,7%) e doença arterial coronariana (41,7%). Todos os pacientes apresentaram sangramento gastrointestinal, porém 50% receberam transfusão de concentrados de hemáceas na admissão. Apenas 33,3% faziam uso de antiagregantes plaquetários (aspirina), e 8,3% de anticoagulação com varfarina. Não houve diferença em relação aos sangramentos com necessidade de transfusão comparando os pacientes em uso de aspirina vs. aqueles sem essa medicação (62,5 vs. 43,8%, respectivamente; p = 0,385), assim como em relação ao uso de varfarina, já que os únicos dois pacientes em uso dessa medicação não apresentaram novos sangramentos.

**Tabela 1:** – Características clínicas, laboratoriais e ecocardiográficas basais da população estudada

	Total (n=24)	Intervenção (n=17)	Clínico (n=7)	p
**Características clínicas**				
Idade (anos)	77 [70-82]	76 [68-81]	82 [81-87]	0,069
Sexo feminino, n (%)	12 (50,0)	7 (41,2)	5 (71,4)	0,371
Diabetes mellitus, n (%)	10 (41,7)	7 (41,2)	3 (42,9)	1,000
Hipertensão, n (%)	19 (79,2)	15 (88,2)	4 (57,1)	0,126
Fibrilação atrial, n (%)	4 (16,7)	3 (17,6)	1 (14,3)	1,000
Doença arterial coronariana, n (%)	10 (41,7)	8 (47,1)	2 (8,6)	0,653
EuroSCORE II (%)	1,78 [1,44-3,42]	1,71 [1,31-2,81]	2,41 [1,57-7,55]	0,371
STS (%)	2,43 [1,60-4,42]	2,21 [1,49-3,95]	4,8 [1,93-6,04]	1,000
**Sintomas**				
Dispneia (NYHA III/IV), n (%)	14 (58,3)	10 (58,8)	4 (57,1)	1,000
Angina, n (%)	3 (12,5)	3 (17,6)	0 (0)	0,530
Sangramento gastrointestinal com necessidade de transfusão, n (%)	12 (50,0)	9 (52,9)	3 (42,9)	1,000
**Medicações**				
Inibidores da ECA ou BRA, n (%)	10 (41,7)	8 (47,1)	2 (28,6)	0,653
Betabloqueadores, n (%)	6 (25,0)	4 (23,5)	2 (28,6)	1,000
Antiagregantes plaquetários, n (%)	8 (33,3)	5 (29,4)	3 (42,9)	0,647
Diuréticos, n (%)	17 (70,8)	11 (64,7)	6 (85,7)	0,625
Estatinas, n (%)	9 (37,5)	6 (35,3)	3 (42,9)	1,000
Digoxina, n (%)	3 (12,5)	2 (11,8)	1 (14,3)	1,000
Anticoagulação oral, n (%)	2 (8,3)	2 (11,8)	0 (0)	1,000
**Parâmetros ecocardiográficos**				
Gradiente transaórtico médio, mmHg	49 [42-57]	53 [42-57]	42 [39-59]	0,141
Velocidade de pico aórtica, m/s	4,5 [4,0-4,9]	4,5 [4,0-5,1]	4,4 [4,0-4,8]	0,381
Fração de ejeção do ventrículo esquerdo, %	64 [56-68]	64 [55-68]	65 [55-69]	
Área valvar aórtica, cm^2^	0,66 [0,60-0,70]	0,66 [0,60-0,70]	0,66 [0,60-0,70]	1,000
Insuficiência aórtica moderada, n (%)	2 (8,3)	1 (5,9)	1 (14,3)	0,507
Insuficiência mitral funcional moderada/importante, n (%)	7 (29,2)	5 (29,4)	2 (28,6)	1,000
Insuficiência tricúspide moderada/importante, n (%)	3 (12,5)	1 (5,9)	2 (28,6)	0,194
**Dados laboratoriais**				
Hemoglobina, g/dL	10,1 [8,2-11,4]	10,4 [8,5-12,6]	9,1 [6,3-11]	1,000
Hemoglobina < 7,0 g/dL na admissão, n (%)	4 (17,4)	1 (6,3)	3 (42,9)	0,067
Hemoglobina < 9,0 g/dL na admissão, n (%)	8 (34,8)	5 (51,3)	3 (42,9)	0,657
Hematócrito, %	32 [25-34]	32 [27-36]	27 [22-33]	0,657
Plaquetas, /mm^3^	210 000 [155 000-281 000]	208 000 [149 000-267 500]	270 000 [175 000-299 000]	0,667
Tempo de protrombina, seg	15,3 [13,2-16,2]	14,6 [12,7-16,1]	15,9 [15,3-17,9]	0,193
Atividade protrombínica, %	78,7 [71,8-92,0]	83,6 [73,5-92,0]	71,8 [63,0-89,8]	0,371
Relação de tempos (TP/TR)	1,1 [1,0-1,2]	1,0 [1,0-1,1]	1,2 [1,0-1,2]	0,650
RNI	1,1 [1,0-1,2]	1,0 [1,0-1,1]	1,2 [1,0-1,3]	0,137
TTPA, seg	29,5 [28,2-33,9]	29,3 [28,3-31,]	30,0 [28,1-36,5]	0,361
Clearance de creatinina, mL/min/1,73m^2^	59 [48-75]	61 [52-70]	28 [28-77]	1,000
Ureia, mg/dL	50 [41-70]	47 [38-56]	71 [50-138]	0,193

*BRA: bloqueador do receptor da angiotensina; ECA: enzima conversora da angiotensina; NYHA: New York Heart Association; RNI: razão normalizada internacional; TP: tempo de protrombina; TR: tempo de reptilase; TTPA: tempo de tromboplastina parcial ativada.*

Todos os pacientes eram portadores de estenose aórtica de etiologia degenerativa, com mediana de gradiente transaórtico médio de 49 [42-57] mmHg, área valvar aórtica de 0,66 [0,60-0,70] cm^2^ e velocidade de pico de 4,5 [4,0-4,9] m/s. A mediana da fração de ejeção do ventrículo esquerdo foi de 64 [56-68] %. O restante das características ecocardiográficas está descrito na Tabela 1 , assim como os dados laboratoriais. A hemoglobina basal era de 10,1 [8,2-11,4] g/dL, e 17,4% e 34,8% dos pacientes apresentavam hemoglobina < 7,0 e < 9,0 mg/dL na admissão, respectivamente. Os valores do coagulograma eram normais, com exceção de um paciente em anticoagulação oral com razão normalizada internacional (RNI) de 1,7. Em relação à colonoscopia e à endoscopia digestiva alta, os dados basais estão descritos na Tabela 2 . Todos os pacientes apresentavam angiodisplasia, 50,0% diagnosticada na colonoscopia, 33,3% na endoscopia digestiva alta, e 16,6% em ambas. As lesões foram mais frequentemente encontradas no cólon ascendente (62,5%) e no estômago (37,5%). Tratamento das lesões com cauterização ou argônio ocorreu em 41,7% dos casos.

**Tabela 2:** – Características da colonoscopia e endoscopia digestiva alta

	Total (n=24)	Intervenção (n=17)	Clínico (n=7)	p
Angiodisplasia, n (%)*				
Cólon ascendente, n (%)	15 (62,5)	9 (52,9)	6 (85,7)	0,191
Cólon transverso, n (%)	2 (8,3)	0 (0)	2 (28,6)	0,076
Estômago, n (%)	9 (37,5)	7 (41,2)	2 (28,6)	0,669
Cólon descendente, n (%)	4 (16,7)	1 (5,9)	3 (42,9)	0,059
Duodeno, n (%)	5 (20,8)	4 (23,5)	1 (14,3)	1,000
Jejuno, n (%)	4 (16,7)	2 (11,8)	2 (28,6)	0,552
Divertículos, n (%)	13 (54,2)	8 (47,1)	5 (71,4)	0,386
Pólipos, n (%)	8 (3,3)	7 (41,2)	1 (14,3)	0,352
Cauterização ou aplicação de argônio, n (%)	10 (41,7)	9 (52,9)	1 (14,3)	0,172

*A soma pode ser maior que 100% pois pacientes podem apresentar mais do que uma localidade da angiodisplasia.

### Intervenção valvar vs. tratamento clínico

Os dados relacionados à intervenção e a eventos estão descritos na Tabela 3 . Em nosso estudo, sete (29,2%) pacientes foram mantidos em tratamento clínico, e 17 (70,8%) foram submetidos à intervenção valvar aórtica, sendo que em 70,5% foi implantada prótese biológica, em 17,6%; prótese mecânica; em 11,7%, implante transcateter de bioprótese aórtica (TAVR); e, em 23,5%, foi realizada revascularização miocárdica concomitante. A mortalidade em 30 dias e 1 ano do grupo intervenção foi de 11,8% e 23,5%, respectivamente. Não encontramos diferenças relacionadas às características basais, laboratoriais, ecocardiográficas nem relacionadas à endoscopia digestiva alta e colonoscopia na comparação entre os grupos (Tabelas 1 e 2). Além disso, a taxa de novos sangramentos foi semelhante nos grupos de tratamento clínico vs. intervencionista (28,6 vs. 23,5%, respectivamente; p=1,000). Dos pacientes do grupo intervenção que apresentaram novos sangramentos gastrointestinais, dois deles necessitaram transfusão de concentrado de hemáceas, e em nenhum desses pacientes a prótese mecânica havia sido implantada. A indicação da intervenção valvar foi presença de sintomas em 15 (88,2%) casos e sangramentos com necessidade de transfusão de concentrados de hemáceas em dois (11,7%) casos. Os óbitos em 1 ano foram decorrentes de: endocardite infecciosa em um caso, sepse em um caso, e choque cardiogênico em dois casos.

**Tabela 3:** – Características da intervenção e eventos em 30 dias

	Intervenção (n=17)	Clínico (n=7)	p
Prótese biológica, n (%)	12 (70,5)	-	-
Prótese mecânica, n (%)	3 (17,6)	-	-
Revascularização miocárdica concomitante, n (%)	4 (23,5)	-	-
TAVR, n (%)	2 (11,7)		
Mortalidade em 30 dias, n (%)	2 (11,8)	0 (0)	1,000
Mortalidade em 1 ano, n (%)	4 (23,5)	0 (0)	0,283
Acidente vascular cerebral incapacitante, n (%)	1 (5,9)	-	-
Derrame pericárdico, n (%)	3 (17,6)	-	-
Fibrilação atrial, n (%)	6 (35,3)	-	-
Reabordagem, n (%)	2 (11,8)	-	-
Sangramento gastrointestinal, n (%)	4 (23,5)	2 (28,6)	1,000
Sangramento gastrointestinal com necessidade de transfusão, n (%)	2 (11,8)	2 (28,6)	0,552

*TAVR indica implante de bioprótese aórtica transcateter, do inglês transcatheter aortic valve replacement.*

Três pacientes do grupo intervenção foram submetidos a nova colonoscopia devido a recorrência de sangramento, sendo evidenciada manutenção das lesões angiodisplásicas nas mesmas localidades encontradas antes da intervenção valvar.

A indicação do manejo clínico medicamentoso em sete (29,2%) pacientes foi devido à escolha do paciente e/ou do médico assistente em função da idade, da fragilidade clínica e de outras comorbidades. A mediana do seguimento após diagnóstico da síndrome de Heyde foi de 24 [12-54] meses, e a mortalidade global no seguimento de 2 e 5 anos foi de 16% e 25%, respectivamente, sem diferença entre os grupos (log-rank p = 0,185 e 0,737, respectivamente).

Para avaliar o impacto do longo intervalo de inclusão, dividimos os pacientes de acordo com a mediana de inclusão: 2005-2010 (n=13) e 2011-2018 (n=11) (Tabelas Suplementares 1 e 2). Apenas a dosagem de ureia apresentou diferença significativa entre os grupos 2005-2010 e 2011-2018 (67 [51-87] vs. 44 [38-49] mg/dL, respectivamente; p=0,012). Não encontramos diferenças em relação às outras características basais, bem como em relação aos desfechos principais de mortalidade em 1 ano (15,4 vs. 18,2 %, respectivamente; p=1,000) e taxas de novos sangramentos (30,8 vs. 18,2%, respectivamente; p=0,649). Houve uma tendência a menor indicação de tratamento clínico isolado no grupo 2011-2018 (46,2 vs. 9,1 %, respectivamente; p=0,078), porém sem significância estatística.

## Discussão

Os principais achados do presente estudo são: 1) 50% dos pacientes apresentaram sangramento gastrointestinal com necessidade de transfusão de hemáceas na admissão, demonstrando que a síndrome de Heyde é uma doença com potencial risco de morte; 2) não houve diferença nas taxas de ressangramento comparando pacientes submetidos ao tratamento intervencionista vs. tratamento clínico medicamentoso; 3) a sobrevida foi similar entre os grupos, inclusive no seguimento tardio; 4) o local de sangramento mais frequente foi o cólon ascendente.

A síndrome de Heyde é definida pela presença de estenose aórtica importante associada a sangramento gastrointestinal decorrente de angiodisplasias e, desde sua primeira descrição em 1958, sua fisiopatogênese não foi completamente elucidada.^1^ Vasos angiodisplásicos são as anormalidades vasculares mais comumente encontradas no trato gastrointestinal, e sua prevalência certamente aumenta com a idade, assim como a prevalência da estenose aórtica.^2 , 4 , 11 , 13^ Entretanto, a correlação entre estenose aórtica importante e a presença de lesões angiodisplásicas não parece ser casual. A obstrução da via de saída do ventrículo esquerdo reduz o fluxo gastrointestinal devido ao achatamento dos padrões de onda dos pulsos arteriais. Tal alteração levaria a isquemia esplâncnica relativa, favorecendo assim a neoangiogênese.^4 , 14 - 17^ Essa hipótese é corroborada por Greensteins et al.,^14^ que estudaram a configuração da onda de pulso arterial na vasculatura mesentérica em pacientes com estenose aórtica, e por Stern et al.,^15^ que avaliaram 33 pacientes com dispositivos de assistência ventricular não pulsátil e encontraram sangramentos decorrentes de angiodisplasias do intestino delgado em 13 deles. Além disso, a mucosa colônica isquêmica torna-se frágil, aumentado o risco de sangramento. Entretanto, o tratamento intervencionista da estenose aórtica não resolve as lesões angiodisplásicas, assim como documentado em três dos nossos casos, em que foi repetida a colonoscopia e/ou endoscopia digestiva alta após a intervenção valvar.^2^ Ademais, o cólon ascendente foi o local onde as lesões foram mais frequentemente encontradas, assim como descrito na literatura.^12^

Outro fator que contribui para o sangramento das lesões angiodisplásicas é a deficiência adquirida do vWF. Os vWF são glicoproteínas multiméricas que se ligam ao fator VIII, contribuindo para a formação do trombo plaquetário e agindo como um mediador da adesão plaquetária.^2 , 3 , 6 , 18 , 19^ Em situações de estresse de cisalhamento decorrente da estenose valvar aórtica, o vWF altera sua estrutura, ocorrendo proteólise desses multímeros mediada pela enzima *A Disintegrin And Metalloproteinase with Thrombospondin type 1 motif, member 13* (ADAMTS13).^2 , 3 , 6 , 18 , 19^ Alguns autores sugerem que a intervenção valvar aórtica (cirúrgica ou transcateter) corrige tal deficiência do vWF, e drasticamente reduzindo a taxa de novos sangramentos.^5 , 6 , 11 , 12 , 19 - 21^ Thompsom et al.,^12^ em uma coorte de 34 anos, avaliaram 57 pacientes com síndrome de Heyde submetidos a troca valvar aórtica cirúrgica e demonstraram que 79% deles não apresentaram novos episódios de sangramento após a intervenção valvar após seguimento com mediana de 4,4 [0,1-15] anos. Os pacientes apresentavam características basais semelhantes às do nosso estudo, porém não havia grupo controle. Em nossa coorte de 13 anos e com mediana de seguimento de 2 [1-4,5] anos, 76,5% dos pacientes também não apresentaram novos episódios de sangramento após a intervenção valvar aórtica. Entretanto, não houve diferença quando estes foram comparados aos pacientes mantidos em tratamento clínico medicamentoso, sugerindo que a intervenção teria o mesmo impacto que o tratamento clínico em relação a novos episódios hemorrágicos em pacientes com síndrome de Heyde.

A síndrome de Heyde não é descrita nas diretrizes atuais de tratamento das doenças valvares. Entretanto, devido à sugestão de resolução do sangramento após a correção da estenose aórtica, alguns autores advogam a indicação de intervenção valvar em pacientes com essa condição, independente da presença de sintomas ou complicadores, assim como realizado em dois pacientes do presente estudo.^11 , 12 , 19 - 21^ Um dado a favor da indicação de intervenção pela síndrome de Heyde é que esta pode ocasionar episódios com alto risco de mortalidade, assim como no estudo atual, em que metade dos pacientes necessitou de transfusão de concentrado de hemáceas na admissão. Em situações de anemia aguda, aqueles com estenose aórtica importante são incapazes de aumentar o débito cardíaco, devido à obstrução fixa da via de saída, sendo assim suscetíveis a eventos cardiovasculares, como insuficiência cardíaca e infarto do miocárdio tipo 2. Apesar disso, 29,2% persistiram em tratamento clínico medicamentoso devido à escolha do paciente e do médico assistente. Pacientes do grupo clínico apresentavam uma tendência a serem mais idosos, porém sem outras diferenças estatisticamente significantes relacionadas às características basais. É importante salientar que tais pacientes foram acompanhados em um hospital terciário do sistema público de saúde brasileiro, no qual o TAVR não era uma opção terapêutica disponível no momento, justificando assim a alta taxa de tratamento conservador e os poucos casos de TAVR.

## Limitações

O estudo atual sofre das limitações inerentes ao seu desenho. Um ponto importante é o tamanho da série que, apesar de relativamente pequena, até o presente momento é a maior série de casos de síndrome de Heyde da América Latina. Apesar disso, estamos sujeitos a erro tipo II para assegurar que o tratamento intervencionista não seja suficiente para a redução de eventos/novos sangramentos. Além disso, foi necessário longo tempo de inclusão, por se tratar de doença rara; todavia, a análise em relação ao tempo de inclusão não mostrou diferenças significativas. Outra limitação é o fato de que alguns aspectos, como fragilidade, pressão sistólica de artéria pulmonar, não puderam ser avaliados, devido às características do estudo, assim como não houve a avaliação da deficiência do vWF. Além disso, pacientes incluídos nos últimos anos tiveram menor seguimento, o que pode impactar a análise de sobrevida em longo prazo, apesar do ajuste estatístico pelo tempo de seguimento.

## Conclusão

Em nossa coorte, pacientes com síndrome de Heyde tiveram alta taxa de sangramento com necessidade de transfusão sanguínea na admissão, sugerindo ser uma doença grave e com risco elevado de mortalidade. Não encontramos diferenças entre os grupos clínico e intervenção em relação a taxas de ressangramento e mortalidade tardia. Estudos prospectivos são necessários para confirmar se a intervenção valvar reduz a taxa de novos sangramentos e se a síndrome de Heyde deveria, por si só, ser indicativa de intervenção valvar. Entretanto, a raridade dessa entidade dificulta a realização de tais trabalhos.
