# Supplementary material for: Síndrome de Heyde: Estratégias Terapêuticas e Seguimento de Longo Prazo
Source: Arq Bras Cardiol. 2021 Jun 25;117(3):512–7. [Article in Portuguese] doi: 10.36660/abc.20200371 (PMC8462952; doi:10.36660/abc.20200371)
Supplement: Translation

# Translation: Heyde’s Syndrome: Therapeutic Strategies and Long-Term Follow-Up

**Authors:** Vitor Emer Egypto Rosa, Henrique Barbosa Ribeiro, João Ricardo Cordeiro Fernandes, Antonio de Santis, Guilherme Sobreira Spina, Milena Ribeiro Paixão, Lucas José Tachotti Pires, Marcelo Bettega, Tarso Augusto Duenhas Accorsi, Roney Orismar Sampaio, Flávio Tarasoutchi

**Affiliations:** 1 Brazil Instituto do Coração do Hospital das Clinicas da Faculdade de Medicina da Universidade de São Paulo, São Paulo, SP - Brazil

**Keywords:** Aortic Stenosis, Angiodysplasia, Hemorrhage, Mortality, Thoracic Surgery, Echocardiography/methods

## Abstract

**Background:**

Heyde’s syndrome is the association of severe aortic stenosis with episodes of gastrointestinal bleeding due to angiodysplastic lesion. Little is known about the factors associated with new episodes of bleeding and long-term outcomes. Furthermore, most data are restricted to case reports and small case series.

**Objective:**

To assess the clinical, laboratory and echocardiography profile of patients with Heyde’s syndrome who underwent valve intervention or drug therapy.

**Methods:**

Prospective cohort of 24 consecutive patients from 2005 to 2018. Clinical, laboratory and echocardiography data were assessed, as well as those related to valve intervention and outcomes after diagnosis. A *P* <0.05 was used to indicate statistical significance.

**Results:**

Half of the 24 patients presented with bleeding requiring blood transfusion on admission. Angiodysplasias were more frequently found in the ascending colon (62%). Valve intervention (surgical or transcatheter) was performed in 70.8% of the patients, and 29.2% remained on drug therapy. News episodes of bleeding occurred in 25% of the cases, and there was no difference between clinical and intervention groups (28.6 vs 23.5%, p = 1.00; respectively). Mortality at 2-year and 5-year was 16% and 25%, with no difference between the groups (log-rank p = 0.185 and 0.737, respectively).

**Conclusions:**

Patients with Heyde’s syndrome had a high rate of bleeding requiring blood transfusion on admission, suggesting that it is a severe disease with high mortality risk. No difference was found between clinical and intervention group regarding the rate of rebleeding and late mortality.

## Introduction

The association between gastrointestinal bleeding due to angiodysplastic lesions and severe aortic stenosis is named Heyde's syndrome and was described by Edward Heyde in 1958.^1^ Since then, there have been controversies on the pathogenesis of this syndrome, and the only factor that predisposes to bleeding in this population is acquired von Willebrand factor (vWF) deficiency resulting from hemodynamic stress in the stenotic aortic valve.^2 - 6^

Heyde's syndrome is described in 1.7% of patients with severe aortic stenosis and, despite its prevalence, little is known about its epidemiological aspects. Most publications related to this entity are case reports and small case series.^5^ The indication of valve intervention in these patients follows current guidelines and, although there are no specific recommendations, some centers advocate for intervention to reduce bleeding, regardless of the presence of symptoms or prognostic factors.^7 - 9^ Therefore, the aim of this study was to evaluate clinical, laboratory, and echocardiography aspects, in addition to mortality rates and complications after valve intervention or clinical treatment, in a case series of patients with Heyde's syndrome.

## Methods

This is a prospective cohort that assessed patients with severe aortic stenosis in a tertiary care center from 2005 to 2018. Severe aortic stenosis was defined as the presence of aortic valve area ≤ 1.0 cm^2^ , mean transaortic gradient > 40 mmHg, or peak velocity > 4.0 m/s as measured by the transthoracic echocardiography.^9^ Patients with history of gastrointestinal bleeding and angiodysplasia documented by colonoscopy and/or upper digestive tract endoscopy were selected. Clinical, laboratory and echocardiography data were assessed, as well as those related to the indicated treatment. Exclusion criteria were: presence of other conditions that could justify bleeding (such as gastric ulcer or neoplasms), severe aortic regurgitation, or anatomically severe primary mitral valve disease. Valve intervention was indicated according to physician's choice and followed current guidelines.^9 , 10^ For comparative reasons, we divided patients into two groups, according to the chosen treatment (valve intervention vs drug therapy). We assessed mortality and perioperative complications at 30 days and later through telephone contact. This study was approved by the institutional ethical research committee, and there was a request to waive the requirement for a consent form.

## Statistical analysis

Continuous and categorical variables were presented as median (interquartile range) and frequencies or percentages, respectively. The normality of variable distribution was assessed using the Kolmogorov-Smirnov test. Continuous variables were analyzed with the Mann-Whitney test, and categorical variables with the chi-square test or the Fisher's exact test, as appropriate. Five-year mortality was assessed using the Log-rank test. All tests were two-tailed, and A *P* <0.05 was used to indicate statistical significance.. The SPSS software, version 20 (IBM, Armonk, New York, USA) was used to analyze data.

## Results

A total of 24 patients met inclusion criteria for the study. Baseline characteristics are described in Table 1 . Median age was 77 [70-82] years, 50% of patients were women, and there was a high prevalence of comorbidities such as systemic arterial hypertension (79.2%), diabetes (41.7%), and coronary artery disease (41.7%). All patients presented gastrointestinal bleeding, but 50% received red blood cell transfusion on admission. Only 33.3% were using platelet antiaggregants (aspirin), and 8.3% were on anticoagulation therapy with warfarin. There was no difference between patients using aspirin vs those not using this medication with regard to bleeding requiring transfusion (62.5 vs 43.8%, respectively; p = 0.385). The same was observed for the use of warfarin, since the only two patients using this medication did not have new episodes of bleeding.

**Table 1:** – Clinical, laboratory and echocardiography characteristics of the study population

	Total (N = 24)	Intervention (n = 17)	Clinical (n = 7)	p
**Clinical characteristics**				
Age (years)	77 [70-82]	76 [68-81]	82 [81-87]	0.069
Female sex, n (%)	12 (50.0)	7 (41.2)	5 (71.4)	0.371
Diabetes mellitus, n (%)	10 (41.7)	7 (41.2)	3 (42.9)	1.000
Hypertension, n (%)	19 (79.2)	15 (88.2)	4 (57.1)	0.126
Atrial fibrillation, n (%)	4 (16.7)	3 (17.6)	1 (14.3)	1.000
Coronary heart disease, n (%)	10 (41.7)	8 (47.1)	2 (8.6)	0.653
EuroSCORE II (%)	1.78 [1.44-3.42]	1.71 [1.31-2.81]	2.41 [1.57-7.55]	0.371
STS (%)	2.43 [1.60-4.42]	2.21 [1.49-3.95]	4.8 [1.93-6.04]	1.000
**Symptoms**				
Dyspnea (NYHA III/IV), n (%)	14 (58.3)	10 (58.8)	4 (57.1)	1.000
Angina, n (%)	3 (12.5)	3 (17.6)	0 (0)	0.530
Gastrointestinal bleeding requiring transfusion, n (%)	12 (50.0)	9 (52.9)	3 (42.9)	1.000
**Medications**				
ACE inhibitors or ARB, n (%)	10 (41.7)	8 (47.1)	2 (28.6)	0.653
Beta-blockers, n (%)	6 (25.0)	4 (23.5)	2 (28.6)	1.000
Platelet antiaggregant drugs, n (%)	8 (33.3)	5 (29.4)	3 (42.9)	0.647
Diuretics, n (%)	17 (70.8)	11 (64.7)	6 (85.7)	0.625
Statins, n (%)	9 (37.5)	6 (35.3)	3 (42.9)	1.000
Digoxin, n (%)	3 (12.5)	2 (11.8)	1 (14.3)	1.000
Oral anticoagulation, n (%)	2 (8.3)	2 (11.8)	0 (0)	1.000
**Echocardiography parameters**				
Mean transaortic gradient, mmHg	49 [42-57]	53 [42-57]	42 [39-59]	0.141
Aortic peak velocity, m/s	4.5 [4.0-4.9]	4.5 [4.0-5.1]	4.4 [4.0-4.8]	0.381
Left ventricular ejection fraction, %	64 [56-68]	64 [55-68]	65 [55-69]	
Aortic valve area, cm^2^	0.66 [0.60-0.70]	0.66 [0.60-0.70]	0.66 [0.60-0.70]	1.000
Moderate aortic insufficiency, n (%)	2 (8.3)	1 (5.9)	1 (14.3)	0.507
Moderate/severe functional mitral insufficiency, n (%)	7 (29.2)	5 (29.4)	2 (28.6)	1.000
Moderate/severe tricuspid insufficiency, n (%)	3 (12.5)	1 (5.9)	2 (28.6)	0.194
**Laboratory data**				
Hemoglobin, g/dL	10.1 [8.2-11.4]	10.4 [8.5-12.6]	9.1 [6.3-11]	1.000
Hemoglobin < 7.0 g/dL on admission, n (%)	4 (17.4)	1 (6.3)	3 (42.9)	0.067
Hemoglobin < 9.0 g/dL on admission, n (%)	8 (34.8)	5 (51.3)	3 (42.9)	0.657
Hematocrit, %	32 [25-34]	32 [27-36]	27 [22-33]	0.657
Platelets, /mm^3^	210 000 [155 000-281 000]	208 000 [149 000-267 500]	270 000 [175 000-299 000]	0.667
Prothrombin time, s	15.3 [13.2-16.2]	14.6 [12.7-16.1]	15.9 [15.3-17.9]	0.193
Prothrombin activity, %	78.7 [71.8-92.0]	83.6 [73.5-92.0]	71.8 [63.0-89.8]	0.371
Time ratio (PT/RT)	1.1 [1.0-1.2]	1.0 [1.0-1.1]	1.2 [1.0-1.2]	0.650
INR	1.1 [1.0-1.2]	1.0 [1.0-1.1]	1.2 [1.0-1.3]	0.137
APTT, s	29.5 [28.2-33.9]	29.3 [28.3-31.]	30.0 [28.1-36.5]	0.361
Creatinine clearance, mL/min/1.73m^2^	59 [48-75]	61 [52-70]	28 [28-77]	1.000
Urea, mg/dL	50 [41-70]	47 [38-56]	71 [50-138]	0.193

*ACE: angiotensin-converting enzyme; APTT: activated partial thromboplastin time; ARB: angiotensin receptor blocker; IRN: international normalized ratio; NYHA, New York Heart Association; PT: prothrombin time; RT: reptilase time.*

All patients had degenerative aortic stenosis, with median mean transaortic gradient of 49 [42-57] mmHg, valve aortic area of 0.66 [0.60-0.70] cm^2^, and peak velocity of 4.5 [4.0-4.9] m/s. Median left ventricular ejection fraction was 64 [56-68]%. Other echocardiography characteristics are described in Table 1 , as well as laboratory data. Baseline hemoglobin was 10.1 [8.2-11.4] g/dL, and 17.4% and 34.8% of patient had hemoglobin < 7.0 and < 9.0 mg/dL on admission, respectively. Results for the coagulogram test were normal, except for one patient on oral anticoagulant therapy with international normalized ratio (INR) of 1.7. With regard to colonoscopy and upper digestive tract endoscopy, baseline data are described in Table 2 . All patients had angiodysplasia, of which 50.0% were diagnosed in colonoscopy, 33.3% in upper digestive tract endoscopy, and 16.6% in both. Lesions were most frequently found in the ascending colon (62.5%) and in the stomach (37.5%). Treatment of lesions with cauterization or argon occurred in 41.7% of the cases.

**Table 2:** – Findings from colonoscopy and upper digestive tract endoscopy

	Total (n=24)	Intervention (n = 17)	Clinical (n = 7)	p
Angiodysplasia, n (%)*				
Ascending colon, n (%)	15 (62.5)	9 (52.9)	6 (85.7)	0.191
Transverse colon, n (%)	2 (8.3)	0 (0)	2 (28.6)	0.076
Stomach, n (%)	9 (37.5)	7 (41.2)	2 (28.6)	0.669
Descending colon, n (%)	4 (16.7)	1 (5.9)	3 (42.9)	0.059
Duodenum, n (%)	5 (20.8)	4 (23.5)	1 (14.3)	1.000
Jejunum, n (%)	4 (16.7)	2 (11.8)	2 (28.6)	0.552
Diverticuli, n (%)	13 (54.2)	8 (47.1)	5 (71.4)	0.386
Polyps, n (%)	8 (3.3)	7 (41.2)	1 (14.3)	0.352
Cauterization or application of argon, n (%)	10 (41.7)	9 (52.9)	1 (14.3)	0.172

*The sum may be higher than 100% because the patients may present with angiodysplasia at more than one site.

### Valve intervention vs drug therapy

Data related to intervention and to events are described in Table 3 . In our study, seven (29.2%) patients remained on clinical treatment, and 17 (70.8%) underwent aortic valve intervention, of which 70.5% received a biological prosthesis, 17.6%, a mechanical prosthesis; 11.7%, transcatheter aortic valve replacement (TAVR), and 23.5% underwent concomitant myocardial revascularization. Thirty-day and 1-year mortality in the intervention group was 11.8% and 23.5%, respectively. No differences were found between the groups with regard to baseline, laboratory and echocardiography characteristics or with regard to findings from upper digestive tract endoscopy and colonoscopy (Tables 1 and 2). Furthermore, the rate of new episodes of bleeding was similar between clinical treatment vs intervention group (28.6 vs 23.5%, respectively; p = 1.000). Of the patients in the intervention group who had new episodes of gastrointestinal bleeding, two required red blood cell transfusion, and none of these patients had received the mechanical prosthesis. Valve intervention was indicated due to presence of symptoms in 15 (88.2%) cases, and due to bleeding requiring red blood cell transfusion in two (11.7%) cases. Deaths at 1 year resulted from infectious endocarditis in one case, sepsis in one case, and cardiogenic shock in two cases.

**Table 3:** – Characteristics of intervention and events at 30 days

	Intervention (n = 17)	Clinical (n = 7)	p
Biological prosthesis, n (%)	12 (70.5)	-	-
Mechanical prosthesis, n (%)	3 (17.6)	-	-
Concomitant myocardial revascularization, n (%)	4 (23.5)	-	-
TAVR, n (%)	2 (11.7)		
30-day mortality, n (%)	2 (11.8)	0 (0)	1.000
1-year mortality, n (%)	4 (23.5)	0 (0)	0.283
Major stroke, n (%)	1 (5.9)	-	-
Pericardial effusion, n (%)	3 (17.6)	-	-
Atrial fibrillation, n (%)	6 (35.3)	-	-
Reintervention, n (%)	2 (11.8)	-	-
Gastrointestinal bleeding, n (%)	4 (23.5)	2 (28.6)	1.000
Gastrointestinal bleeding requiring transfusion, n (%)	2 (11.8)	2 (28.6)	0.552

*TAVR indicates transcatheter aortic valve replacement.*

Three patients in the intervention group underwent another colonoscopy due to bleeding recurrence, which shown that the angiodysplastic lesions were still located at the same sites where they were found before valve intervention.

The indication for drug therapy in seven (29.2%) patients was made according to either patient's or assisting physician's choice due to age, frailty, and other comorbidities. Median follow-up after diagnosis of Heyde's syndrome was 24 [12-54] months, and overall mortality at 2-year and 5-year follow-up was 16% and 25%, respectively, with no difference between the groups (log-rank p = 0.185 and 0.737, respectively).

In order to assess the impact of the long period of study inclusion, patients were divided according to the median period of study inclusion: 2005-2010 (n = 13) and 2011-2018 (n = 11) (Supplemental Tables 1 and 2). Only urea levels showed a significant difference between the 2005-2010 and 2011-2018 groups (67 [51-87] vs 44 [38-49] mg/dL, respectively; p = 0.012), No differences were found with regard to other baseline characteristics, as well as with regard to 1-year mortality (15.4 vs 18.2 %, respectively; p = 1.000) and rates of new episodes of bleeding (30.8 vs 18.2%, respectively; p = 0.649). There was a trend of fewer indications of isolated drug therapy in the 2011-2018 group (46.2 vs 9.1 %, respectively; p = 0.078), but with no statistical significance.

## Discussion

The main findings of the present study were: 1) 50% of the patients presented with gastrointestinal bleeding requiring red blood cell transfusion on admission, showing that Heyde's syndrome is a potential life-threatening disease; 2) there was no difference in the rates of rebleeding between patients that underwent interventional treatment vs drug therapy; 3) survival was similar between the groups, inclusive no in long-term follow-up; 4) the most frequent bleeding site was the ascending colon.

Heyde's syndrome is defined by the presence of severe aortic stenosis associated with gastrointestinal bleeding resulting from angiodysplasias and, since it was first described in 1958, its pathophysiogenesis has not been completely elucidated yet.^1^ Angiodysplastic vessels are the most commonly found vascular anomaly in the gastrointestinal tract, and its prevalence certainly increases with age, as well as aortic stenosis prevalence.^2 , 4 , 11 - 13^ However, the correlation between severe aortic stenosis and angiodysplastic lesions does not seem to be casual. Left ventricular outflow tract obstruction reduces gastrointestinal blood flow, due to flattening of arterial pulse wave parameters. This change would lead to relative splanchnic ischemia, thus favoring neoangiogenesis.^4 , 14 - 17^ This hypothesis is corroborated by Greensteins et al.,^14^ who studied the configuration of the pulse wave in the mesenteric vessel in patients with aortic stenosis, and by Stern et al.,^15^ who evaluated 33 patients with non-pulsatile ventricular assist devices and observed episodes of bleeding resulting from small bowel angiodysplasias in 13 of them. Furthermore, ischemic colonic mucosa becomes fragile, thus increasing the risk for bleeding. However, interventional treatment of aortic stenosis does not resolve angiodysplastic lesions, as documented in three of our cases, in which colonoscopy and/or upper digestive tract endoscopy was repeated after valve intervention.^2^ Moreover, ascending colon was the most frequent site of angiodysplastic lesions, in line with the literature.^12^

Other factor contributing to bleeding of these lesions is acquired vWF deficiency. vWFs are multimeric glycoproteins that bind to factor VIII, contributing to formation of platelet thrombus and acting like a mediator of platelet adhesion.^2 , 3 , 6 , 18 , 19^ In situations of shear stress due to aortic valve stenosis, vWFs changes their structure, being subjected to proteolysis mediated by the A Disintegrin and Metalloproteinase with Thrombospondin type 1 motif, member 13 (ADAMTS13) enzyme.^2 , 3 , 6 , 18 , 19^ Some authors suggest that aortic valve intervention (surgical or transcatheter) corrects vWF deficiency, drastically reducing the rate of new bleeding episodes.^5 , 6 , 11 , 12 , 19 - 21^ Thompsom et al.^12^ assessed a 34-year cohort of 57 patients with Heyde's syndrome who underwent surgical aortic valve replacement and showed that 79% of patients did not experience new episodes of bleeding after valve intervention at a median follow-up of 4.4 [0.1-15] years. These patients had baseline characteristics similar to those of patients included in our study, but there was no control group. In our 13-year cohort with a median follow-up of 2 [1-4.5] years, 76.5% of patients did not experience new episodes of bleeding after aortic valve intervention, but there was no difference between these patients and those who remained on drug therapy, suggesting that intervention would have the same impact as drug therapy with regard to new bleeding episodes in patients with Heyde's syndrome.

Heyde's syndrome is not described in current guidelines for the management of patients with valvular heart disease. Nevertheless, due to the hypothesis that bleeding may be resolved after aortic stenosis intervention, some authors advocate for the indication of valve intervention in patients with this condition, regarding of the presence of symptoms or prognostic factors, as two patients in the present study.^11 , 12 , 19 - 21^ Indication for intervention due to Heyde's syndrome is favored by the fact that this disease may lead to episodes posing high mortality risk, as it could be observed in the present study, in which half of the patients required red blood cell transfusion on admission. In situations of acute anemia, those with severe aortic stenosis are unable to increase cardiac output, due to fixed outflow obstruction, being susceptible to cardiovascular events, such as heart failure and myocardial infarction. Nevertheless, 29.2% remained on drug therapy according to either patient's or assisting physician's choice. Patients in the clinical group tended to be older, but there were no other statistically significant differences related to baseline characteristics. It is important to highlight that these patients were followed in a hospital belonging to the Brazilian public health system, in which TAVR was not a currently available therapeutic option, which justifies the high rates of conservative treatment and the few cases of TAVR.

## Limitations

The current study has limitations inherent to its design. An important aspect is the sample size, which, although being relatively small, is the largest case series of patients with Heyde's syndrome in Latin America so far. However, we are susceptible to type II error to ensure that interventional treatment is not enough to reduce events/new bleeding episodes. Furthermore, a long period of period of study inclusion was necessary, because Heyde's syndrome is a rare disease; however, the analysis with regard to period of study inclusion did not reveal significant differences. Another limitation is the fact that some aspects, such as frailty and pulmonary artery systolic pressure, could not be assessed, due to study characteristics; similarly, vWF deficiency was not assessed either. Moreover, patients included in the last year had a shorter follow-up time, which may have an impact on the analysis of long-term survival, despite statistical adjustment by follow-up time.

## Conclusion

In our cohort, patients with Heyde's syndrome showed a high rate of bleeding requiring blood transfusion at admission, suggesting that it is a severe disease with high risk of mortality. No differences were found between clinical and intervention groups with regard to rates of rebleeding and long-term mortality. Prospective studies are needed to confirm whether valve intervention reduces the rate of new bleeding episodes and whether Heyde's syndrome alone should be a reason to indicate valve intervention. Nevertheless, the rarity of this condition hamper the conduction of these studies.
